# Illness Perceptions in Patients of Schizophrenia: A Preliminary Investigation from Lahore, Pakistan

**DOI:** 10.12669/pjms.334.13128

**Published:** 2017

**Authors:** Sadia Hussain, Nazish Imran, Usman Amin Hotiana, Nauman Mazhar, Aftab Asif

**Affiliations:** 1 Ms. Sadia Hussain, MSc Psychology. Intern Psychologist, Academic Department of Psychiatry & Behavioural Sciences King Edward Medical University/Mayo Hospital, Lahore, Pakistan; 2 Dr. Nazish Imran, MBBS; FRCPsych; MRCPsych (London). Associate Professor, Child & Family Psychiatry Department. King Edward Medical University/Mayo Hospital, Lahore, Pakistan; 3 Dr. Usman Amin Hotiana, MBBS; FCPS (Psy). Assistant Professor, King Edward Medical University/Mayo Hospital, Lahore, Pakistan Academic Department of Psychiatry & Behavioral Sciences; 4 Dr. Nauman Mazhar, MBBS; MD(Psy); FCPS(Psy)Senior Registrar, Department of Psychiatry Services Hospital, Lahore, Pakistan; 5 Prof. Aftab Asif, MBBS; MRCPsych Professor of Psychiatry, Academic Department of Psychiatry & Behavioural Sciences, King Edward Medical University/Mayo Hospital, Lahore, Pakistan

**Keywords:** Schizophrenia, Illness perceptions, IPQ, compliance, self-regulation

## Abstract

**Background and Objective:**

Patient’s perception of their illness influences their healthcare decisions. The objectives of this study were to explore patient’s own beliefs about their illness (Schizophrenia) and perceived social support, and its impact on their attitudes toward pharmacological treatment in Lahore, Pakistan.

**Methods:**

This study was conducted at Mayo Hospital Lahore from March to September 2016. Hundred individuals suffering from Schizophrenia completed four questionnaires; a socio-demographic questionnaire, the Illness Perception Questionnaire for Schizophrenia(IPQ-S), Drug attitude Inventory-10 (DAI) and Multidimensional Scale of Perceived Social Support (PSS).

**Results:**

Stress, family problems, lack of friends & financial worries were endorsed strongly by patients as cause of their mental illness. Ambiguity regarding their mental illness duration and personal control was observed. Patients’ perceived significant negative consequences, negative emotional response, as well as had poor understanding of their mental illness and treatment effectiveness. Statistically significant gender differences in treatment control and illness coherence subscales of IPQS were observed. Drug attitude inventory was positively correlated with Treatment control subscale (p < .01) and negatively correlated with Illness coherence subscale of IPQS (p < .05). The negative consequences subscale and perceived social support was negatively correlated (p < .01).

**Conclusion:**

Patient’s perception about their own illness is predictor of their drug taking attitude and perceived social support. Study results should help to develop new interventions to correct inaccurate beliefs in patients with schizophrenia to improve illness outcome.

## INTRODUCTION

Perception of illness influences treatment and outcome of any disease^1^ and evidence suggests that illness perceptions are also applicable across a wide range of common mental health disorders, including schizophrenia^2^, non-affective psychotic disorder^3^, bipolar disorder^4^ anorexia nervosa^5^, depression^6^ , and anxiety.^7^ Illness perception approach proposes that understanding the way a patient perceives their condition can help in understanding their behaviour and lead to new ways to assist their adjustment.^8^ The work on illness perception is based on “cognitive representations “of illness in self-regulation model proposed by Howard Leventhal. It proposes that individuals form beliefs concerning their illness in order to understand and cope with the health problems.^9^

Illness perceptions have been associated with emotional distress, disability, adherence to the recommended treatment regimen, recovery and engagement in services in Psychosis.^10^,^11^ Thus understanding illness perceptions and incorporating them into health care is critical to improving treatment outcomes in patients with schizophrenia. Much of our knowledge of illness perceptions in various psychiatric illnesses including schizophrenia has been informed by literature from the west but Illness perception (IP) has been shown to vary across countriesand culture.^12^,^13^ Mentally ill patients perceptions and experiences of their illness in Asian cultures has largely been neglected.

With this knowledge gap in mind, the objective of the study was to encourage understanding of Patient’s own beliefs about their illness and its impact on their attitudes toward pharmacological treatment in patients with schizophrenia in Lahore, Pakistan.

## METHODS

This study was conducted at Mayo Hospital Lahore from March to September 2016. Ethical approval for the study was granted by the Institutional Review Board of King Edward Medical University/Mayo Hospital Lahore. The study was conducted with two hundred participants, (100 patients and 100 primary caregivers of these patients). This paper is focused on Patients illness perception solely and its correlation with drug attitudes and perceived social support. Criteria for patients inclusion was age between18-65, informed consent, meeting DSM-V criteria for Schizophrenia and stable mental state for at least four weeks prior to the data collection. People with primary diagnosis of a learning disability or substance abuse were excluded.

Each participant was verbally explained the nature of the research and confidentiality was assured and maintained. Following written informed consent, participants filled out following self-administered questionnaire in Urdu Language. Socio-demographic Questionnaire (age, gender, occupation, duration of illness and prescribed medications).

Illness representations were measured using ‘Illness Perception Questionnaire for Schizophrenia”.^14^ IPQ-S has various subscales reflecting different dimensions of illness perceptions; Causes, timeline (beliefs in a chronic and relapsing course), timeline cyclical (cyclical nature of illness), consequences(impact of illness on patients life), personal control(patient’s perception of their own control of their illness), treatment control (controllability of illness by treatment), illness coherence (patient’s own understanding of their illness)and emotional representations (feeling of emotions like sadness, anger about their illness). Each statement is rated on 5 point Likert scale (ranging from 1=strongly disagree to 5=strongly agree). Addition of scores of various items yield subscale scores except causes in which individual items are scored as such.

The Drugs Attitude was measured with Drug attitude Inventory (DAI).^15^ It is a self-administered scale developed to measure subjective response and attitudes towards prescribed medication and correlates highly with adherence. DAI is a 10-item true/false scale; a correct answer scored as +1, and an incorrect answer scored as −1. The final DAI score is the sum of the pluses and minuses. A positive total score indicates a positive attitude towards medication, and a negative total score, a negative attitude.

Multidimensional Scale of Perceived Social Support (MPSS)^16^ was used to assess of perceived social support from family and friends. It is a 12-item scale divided into factor groups relating to the source of the social support, namely family (Fam), friends (Fri) or significant other (SO). Each item is scored 1-7 (Very Strongly Disagree = 1- Very Strongly Agree = 7). Possible range for total is 7-84 with categories of High, Moderate and Low Acuity.

Data was analyzed using SPSS 17 version. Descriptive statistics were used to describe socio economic characteristics and IPQR-S subscales, DAI-10 and MPSS. To test associations between scales and subscales correlation analysis was used. Statistical significance was fixed at level of P<0.05. Multiple regression analysis was done to determine if IPQR-S was independent predictor of DAI-10 and MPSS in the study sample.

## RESULTS

Hundred patients (55 Females and 45 males) were recruited for the study. The mean age of sample was 36.06 years (SD=11.16). Majority of the patients in our sample were married (49%) and living in joint family system (61%). Almost forty five percent of patients had education below matriculation (equivalent to high school); 20% were illiterate and very few (27%) were skilled workers. Most of the patients belonged to urban areas (67%). The average illness duration was 6.40 years(sd=4.60) while average length of contact with mental health services was 4.75 years(sd=3.83) Twenty two percent of the sample were receiving regular depot medications alongside oral treatment and 77% were receiving oral antipsychotic medications(most commonly atypical).

The IPQ-S mean item score (total divided by the number of items), median scores and standard Deviation for each subscale except “cause” subscale is shown in Table-I. Individual items on the cause subscale were ranked in terms of strength of belief. Each item had a possible belief rating from 1 to 5. The most strongly held beliefs of patients reported about the causes of mental health problems (median = 4) were that the mental health problems had been caused by stress or worry, family problems, lack of friends or people who care about me, thinking about things too much, money worries and lack of sleep. The beliefs least likely to be endorsed (median = 1) were that the mental health problems were due to alcohol and taking illicit drugs. Cronbach alpha reliability was high for all measures (IPQS= .78, PPS =.81).

All the other subscales are scored on a scale of 1–5, with 3 representing a ‘neither agree nor disagree’ midpoint. Overall, the sample showed ambiguity regarding their mental health problems as being chronic and cyclical in nature, and having personal control over the problems. They perceived significant negative consequences as a result of having mental health problems. There was a general perception of personal blame for the illness & limited belief in the effectiveness of treatment. High score in illness coherence subscale indicates patient’s sense of not having a coherent understanding of their mental health problems. Finally, there was an overall negative emotional response to having mental health problems. (Table-I)

**Table-I:** Mean scores, median scores for each subscale of IPQS, Drug Attitude Inventory and perceived social support (N=100).

*Variable*	*Mean (SD)*	*Median (Range)*
Illness perception		
Timeline acute/chronic	2.85(.86)	3.00(3.50)
Timeline cyclical	2.69(.61)	2.80(2.80)
Consequences	3.66(.56)	3.72(3.09)
Personal control	2.92(.54)	3.00(2.50)
Personal blame	3.14(.95)	3.16(4.00)
Treatment control	2.69(.61)	2.80(2.80)
Illness coherence	3.35(.67)	3.40(3.20)
Emotional representation	3.22(.57)	3.33(3.00)
Drug Attitude Inventory	.88(3.79)	2.00(18.00)
Perceived Social Support	42.35(12.53)	40.0(62.0)

The independent sample *t*-test showed statistically significant differences between male and female patients in treatment control and illness coherence subscales of IPQS (*p* < .05) with male patients having better treatment effectiveness perceptions as well as illness coherence.(Table-II) Regarding attitudes towards drug treatment, females (M= -.06, SD= 3.38) had more negative attitude towards drug as compared to male patients (*M* = 2.36, *SD* = 3.62) (*p* < .000). There was also significant difference between males and female patients on perceived social support (*p* < .01).

**Table-II:** Gender Differences in IPQS, Drug Attitude and perceive social support (N= 100).

Variable	Male M (SD)	Female M(SD)	*P* Value
IPQ-S			
Timeline acute/chronic	16.8 (5.78)	17.40 (4.57)	0.61
Timeline cyclical	15.56 (3.59)	16.24 (2.90)	0.30
Consequences	40.66 (10.22)	42.98 (7.87)	0.20
Personal control	11.60 (2.24)	11.76 (2.09)	0.71
Personal blame	8.94 (2.65)	9.90 (3.01)	0.09
Treatment control	14.10 (3.24)	12.86 (2.85)	0.04*
Illness coherence	16.10 (3.48)	17.42 (3.16)	0.05*
Emotional representation	29.18 (5.62)	28.82 (4.69)	0.72
Drug Attitude Inventory	2.36 (3.62)	-0.60 (3.38)	0.00***
Perceived Social Support	2.48 (0.67)	2.76 (0.47)	0.01**

*** ***Note***: p<.000,

** p<.01,

* p<.05,

*M*= mean; *SD* = standard deviation.

Treatment control subscale of IPQS has significant positive correlation with DAI (p < .01), indicating that having a stronger belief in ability of treatment to control mental health problems was associated with more positive attitude towards taking medication. Table-III. On the other side, Illness coherence subscale of IPQS was found to be negatively correlated (r = -.23, p < .05) with DAI (drug attitude inventory); Signifying that the perception of not having a coherent understanding of mental health problems was associated with negative attitude towards taking medication. The negative consequences subscale and PSS (perceived social support) was negatively correlated (r = -.69, p < .01) with each other’s emphasizing that having more negative consequences is linked with perceived less social support.

**Table-III:** Correlation between IPQS- subscales, Drug Attitude Inventory and perceived social support (N= 100).

*Variable*	*TC*	*CQ*	*PC*	*PB*	*TC*	*IC*	*ER*	*DAI*	*PSS*
Timeline acute/chronic	0.18	0.11	0.23*	0.05	-0.05	0.37**	0.17	0.02	-0.06
Timeline cyclical		0.18	0.13	0.26**	0.06	0.08	0.12	-0.06	-0.16
Consequences			0.07	0.15	0.02	-0.11	-0.02	-0.08	-0.69**
Personal control				0.05	0.08	0.01	0.04	0.02	-0.02
Personal blame					0.03	-0.24	-0.10	-0.01	-0.12
Treatment control						-0.14	0.01	0.49**	0.04
Illness coherence							0.18	-0.23*	0.09
Emotional representation								-0.10	0.07
Drug Attitude Inventory									0.14
Perceived Social Support									1

***Note:*** Timeline cyclical =TC; Consequences= CQ; Personal control= PC; Personal blame =PB; Treatment control= TC; Illness coherence= IC; Emotional representation= ER; Drug Attitude Inventory= DAI; Perceived Social Support = PSS;

** p<.01,

* p<0.05.

Multiple regression analysis was carried out to find the significant predictors of drug taking attitude and perceived social support. (Table IV and V respectively). Treatment control subscale was found to be an independent predictor of DAI (β = .48, *p*< .000) while illness coherence was a significant negative independent predictor of DAI (β = -.25, *p*< .01). The perception of negative consequence of mental health problems subscale was found to be a strong negative independent predictor of perceived social support in our sample (β = -.68, *p*< .000).

**Table-IV:** Multiple regression coefficients for independent predictors of Patients reported Drug Attitude inventory (DAI).

*Variable*	*B*	*SE*	*B*
Timeline acute/chronic	0.14	0.07	0.19
Timeline cyclical	-0.05	0.10	-0.04
Consequences	-0.05	0.03	-0.12
Personal control	-0.05	0.15	-0.03
Personal blame	-0.10	0.12	-0.08
Treatment control	0.59	0.10	0.48***
Illness coherence	-0.28	0.11	-0.25**
Emotional representation	-0.07	0.06	-0.10
R^2^	0.33		
F	5.61		

*** ***Note:*** P < 0.000,

** P<0.01 (two-tailed).

**Table-V:** Multiple regression coefficients for independent predictors of Patients reported perceived Social Support (PSS).

*Variable*	*B*	*SE*	*B*
Timeline acute/chronic	0.03	0.20	0.01
Timeline cyclical	-0.22	0.31	-0.05
Consequences	-0.93	0.10	-0.68***
Personal control	0.10	0.45	0.01
Personal blame	0.01	0.36	0.004
Treatment control	0.26	0.30	0.06
Illness coherence	0.07	0.32	0.02
Emotional representation	0.15	0.18	0.06
R^2^	0.49		
F	10.99		

*** ***Note:***P < .000,

**P<.01 (two-tailed).

## DISCUSSION

Our study is an important first step in understanding beliefs that individuals hold about mental illness in Pakistan. Previous research has shown that “patients’ perceptions of their illness guide their decisions about health.” i.e. perception of illness influences an individual’s functioning, utilization of health care, adherence to treatment plans and even overall mortality. It has also been suggested that how patients views their illness may play a bigger role in determining patient’s health outcomes than the actual severity of the disease.^10^ These beliefs are associated with various emotional and behavioral responses and their understanding will increase possibility of developing culture specific intervention on challenging negative beliefs. Patients were quite positive about being asked about their views of their mental health problems as it is rarely done in clinical practice. Pollack & Aponte in 2001 observed that illness perceptions questionnaire are on their own therapeutic, in allowing patients to tell their story and clarify aspects of their illnesses.^4^

In common with other studies of illness perceptions in mental illness,^10^,^17^ majority of our study participants reported overall negative cognitive representation of their illness & generally felt that they do not have a coherent model of illness. Looking at the beliefs patients have, about what caused their illness, majority endorsed stress and worrying too much as a causal factor for Psychosis. This is in line with the most widely accepted view of psychosis i.e the stress vulnerability model.^18^ It suggests that varying degree of stress may trigger an underlying vulnerability to develop psychosis in people. It was surprising to note that alcohol and drugs were one of the least endorsed items by our participants despite very high rates of substance abuse reported in patients with Psychosis.^19^ Research has also shown that stress, chemical imbalance, organic disorder (genetic, brain disorder) are often identified by public as the most likely cause of schizophrenia. Biogenetic causal beliefs were observed to increase rejection of people with schizophrenia.^20^ Beliefs about cause’s influences the type of treatment patient seeks, for their condition.

Our findings suggest that patients with schizophrenia showed ambiguity about their illness as being chronic or cyclical in nature. Timeline perceptions are important as it would not make any sense to patient to continue long term treatment, when they perceive their illness to be acute/cyclical in nature.^21^ We observed that our patients endorsed negative consequences of their illness and its disabling nature. However, we did not assess for depression, which may also influence patient to have negative cognitions regarding their illness. Worse consequences scores was found to be associated with poor functioning and quality of life in patients with Psychosis.^2^ Negative illness perceptions and poor insight have also been linked with high unmet needs among mental health service users as well as default of depression treatment in elderly.^22^ In addition, predictors of engagement in treatment in Psychosis reported by Freemanincluded beliefs held by an individual about their difficulties.^17^ Patients who engaged fully in therapy tended to endorse more internal/ psychological attribution of their illness, believed that their illness can be controlled / cured and expected chronic nature of their illness.^23^ Similar results of illness perceptions and treatment engagement have been reported in secure settings.^24^

Results of our study also showed that illness representations influence perceived social support and drug attitudes. Indeed, this is in line with previous studies reporting that patients with schizophrenia are most likely to report experiences of stigma.^25^ The perception of treatment efficacy can influence compliance to a treatment regime.^26^ It is of concern, that almost half of the patients with chronic mental illness are non-compliant with their medications leading to poor outcome.^27^ According to a recent study, “a doctor can make accurate diagnoses and have excellent treatments but if the therapy doesn’t fit with the patient’s view of their illness, they are unlikely to keep taking it”.^10^ Positive correlation between treatment control subscale and DAI scores in our study suggests that people who believe treatment will help them have positive attitude towards medication. Similar correlation was observed by Lobban et al in 2005.^14^ Positive attitudes towards drugs have been reported to highly correlate with actual adherence in schizophrenic patients in many studies.^17^,^28^ Asking patients about their views, concerns and fears regarding treatment gives doctors the opportunity to identify and correct any inaccurate beliefs they may have.

Illness perceptions change over time. It is recommended that in patients with Psychosis, illness perceptions should be assessed when patients are in remission and able to make sense of their illness, as was done in our study.^29^

### Limitations of the study

Participants were recruited from just one hospital in urban setting thus generalizability of findings is limited. Sample size is small. Although the scales shows good internal reliability, but they are not yet validated in Pakistani population. Furthermore scales are self-reported and rely on patients own perception of the problems, which is potentially influenced by poor insight. Cross sectional study design does not allow causal inferences to be drawn from the findings. Qualitative studies will be more helpful to investigate various dimensions about beliefs of mental illness in future.

Nevertheless, despite these limitations, findings of the study are important and consistent with the existing literature on this topic. We believe that this work has potential to develop therapeutic approaches to improve patient’s understanding and adjustment to their illness. Further studies should focus on evaluation of effective interventions that are able to reach a large number of patients and are based on these illness representations.

### Authors Contribution

***SH, NI:*** Conception and design, Acquisition of data, data analysis & interpretation, drafting the manuscript.

***UAH, NM:*** Conception, Literature search, acquisition and analysis of data.

***AA:*** Design, Write-up, critical revision.

All authors havecontributed and have approved the final manuscript.
